# High *BAALC* copy numbers in peripheral blood prior to allogeneic transplantation predict early relapse in acute myeloid leukemia patients

**DOI:** 10.18632/oncotarget.21322

**Published:** 2017-09-27

**Authors:** Madlen Jentzsch, Marius Bill, Juliane Grimm, Julia Schulz, Karoline Goldmann, Stefanie Beinicke, Janine Häntschel, Wolfram Pönisch, Georg-Nikolaus Franke, Vladan Vucinic, Gerhard Behre, Thoralf Lange, Dietger Niederwieser, Sebastian Schwind

**Affiliations:** ^1^ Department of Hematology and Oncology, University of Leipzig, Leipzig, Germany

**Keywords:** acute myeloid leukemia, allogeneic stem cell transplantation, residual disease, *BAALC*, prognosis

## Abstract

High *BAALC* expression levels at acute myeloid leukemia diagnosis have been linked to adverse outcomes. Recent data indicate that high *BAALC* expression levels may also be used as marker for residual disease following acute myeloid leukemia treatment. Allogeneic hematopoietic stem cell transplantation (HSCT) offers a curative treatment for acute myeloid leukemia patients. However, disease recurrence remains a major clinical challenge and identification of high-risk patients prior to HSCT is crucial to improve outcomes. We performed absolute quantification of *BAALC* copy numbers in peripheral blood prior (median 7 days) to HSCT in complete remission (CR) or CR with incomplete peripheral recovery in 82 acute myeloid leukemia patients using digital droplet PCR (ddPCR) technology. An optimal cut-off of 0.14 *BAALC*/*ABL1* copy numbers was determined and applied to define patients with high or low *BAALC*/*ABL1* copy numbers. High pre-HSCT *BAALC*/*ABL1* copy numbers significantly associated with higher cumulative incidence of relapse and shorter overall survival in univariable and multivariable models. Patients with high pre-HSCT *BAALC*/*ABL1* copy numbers were more likely to experience relapse within 100 days after HSCT. Evaluation of pre-HSCT *BAALC*/*ABL1* copy numbers in peripheral blood by ddPCR represents a feasible and rapid way to identify acute myeloid leukemia patients at high risk of early relapse after HSCT. The prognostic impact was also observed independently of other known clinical, genetic, and molecular prognosticators. In the future, prospective studies should evaluate whether acute myeloid leukemia patients with high pre-HSCT *BAALC*/*ABL1* copy numbers benefit from additional treatment before or early intervention after HSCT.

## INTRODUCTION

The identification of cytogenetic, molecular, and clinical factors impacting on outcome at acute myeloid leukemia (AML) diagnosis improved risk stratification [1, 2]. But pre-treatment AML characterization may not capture all parameters important for outcome, e.g. response or resistance to therapy [3]. Early detection of measurable residual disease (MRD) through multiparameter flow cytometric (MFC) or quantitative real time PCR (qRT-PCR) assays may allow treatment intervention before overt relapse occurs [3–5]. MFC enables MRD assessment through detection of aberrant surface antigen expression in complete remission (CR) [Wormann *et al*, ASH 1991, 6, 7]. However, heterogenic outcomes were observed in MFC-MRD studies [8] and reproducibility of MFC-MRD assessment is limited by the need of specialized laboratories [3, 4]. Sensitive qRT-PCR enabled MRD detection in AML cases with common fusion genes and in *NPM1* mutated AML [3, 9, 10]. Thus qRT-PCR MRD monitoring is widely restricted to patients carrying specific molecular alterations [11] with the exception of Wilms' tumor gene 1 (*WT1*) expression [9, 12]. Because clonal evolution can occur at disease progression and might complicate early disease detection at relapse [13], it seems reasonable to track several MRD markers per patient.

The gene brain and acute leukemia, cytoplasmic (*BAALC*) has been suggested as a suitable MRD marker as it is expressed at low levels in peripheral blood and bone marrow of healthy individuals [14, 15], but upregulated in AML patients [15]. High *BAALC* expression levels at AML diagnosis have been shown to associate with adverse outcomes [16–19]. Recently, high *BAALC* levels have also been linked to worse outcome if measured by qRT-PCR after achievement of CR [15], completion of induction therapy [11, 20] or after allogeneic stem cell transplantation (HSCT) [21]. However, qRT-PCR has the disadvantage of the need of calibration curves and poor inter-laboratory comparability. In chronic myeloid leukemia (CML) this led to complex harmonization efforts for *BCR-ABL1* detection [22], which are not yet clinical practice for MRD markers in AML. Here we adopted digital droplet PCR (ddPCR), a new technique which allows an absolute quantification without the need of standard curves [23].

Allogeneic HSCT is a potential curative treatment option for AML patients and offers the highest chance of sustained remissions [2]. Non-myeloablative conditioning regimens (NMA), in which the therapeutic success is mainly based on graft-versus-leukemia (GvL) effects, enabled allogeneic HSCT in comorbid or older individuals [24]. Disease recurrence after HSCT remains a major clinical problem with short patient survival [25]. Until today, no study evaluated the feasibility of *BAALC* expression levels for risk stratification in AML patients prior to allogeneic HSCT in CR or CR with incomplete peripheral recovery (CRi), which was the main objective of our study. Early identification of AML patients at high risk of relapse may result in adjustment of treatment strategies prior to morphologic relapse and subsequently improve outcomes. With the goal of a robust, rapid, and reproducible approach, we used peripheral blood to assess the feasibility of ddPCR for absolute quantification of *BAALC*/*ABL1* copy numbers.

## RESULTS

### BAALC/ABL1 copy numbers in AML patients prior to HSCT and in healthy individuals

**Figure 1:**
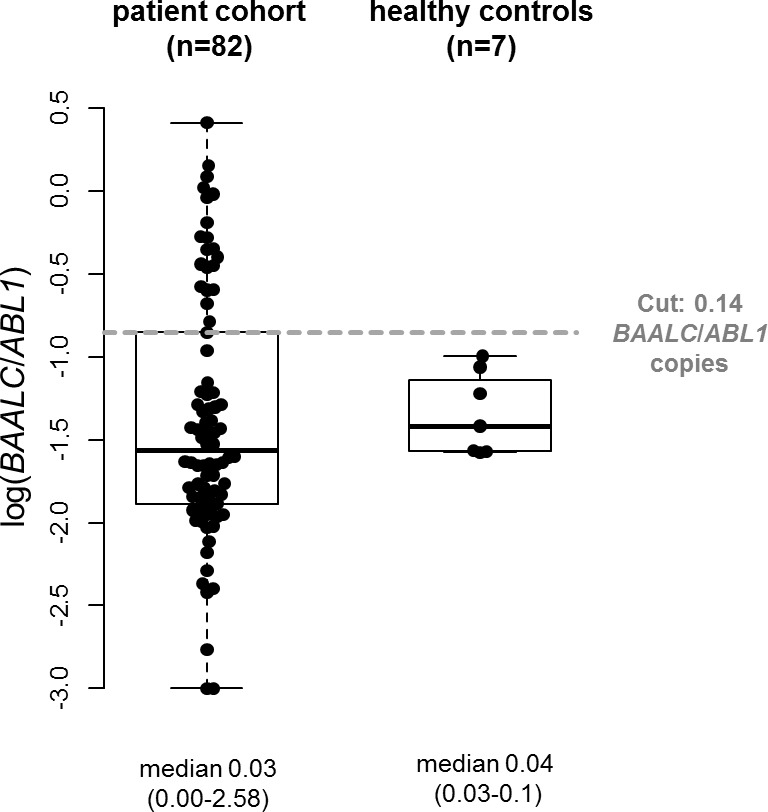
Comparison of absolute *BAALC*/*ABL1* copy numbers in AML patients pre-HSCT (n=82) and healthy controls (n=7)

Within the patient cohort in CR or CRi prior to HSCT, we observed a median pre-HSCT *BAALC*/*ABL1* copy number of 0.03 (range 0.00-2.58, Figure 1). In the healthy control cohort, median *BAALC*/*ABL1* copy numbers were 0.04 (range 0.03-0.10). Overall, there was no significant difference in the *BAALC*/*ABL1* copy numbers between both groups (*P*=.34). The patient cohort and the healthy control cohort were evenly matched in age (*P*=1) and sex (*P*=1, Supplementary Table 2).

### Associations of high pre-HSCT *BAALC/ABL1* copy numbers with clinical and biological characteristics

Patients with high and low pre-HSCT *BAALC*/*ABL1* copy numbers did not differ significantly in the evaluated characteristics at diagnosis (Table 1, Supplementary Table 1). However, there was a trend for a lower incidence of *CEBPA* mutations in patients with high pre-HSCT *BAALC*/*ABL1* copy numbers (*P*=.09). Patients with high and low pre-HSCT *BAALC*/*ABL1* copy numbers also did not differ significantly in pre-HSCT characteristics; specifically, no significant differences were found regarding the remission status at HSCT, white blood count at time of blood sampling for *BAALC*/*ABL1* copy number evaluation or time from blood sampling to HSCT (Supplementary Table 1).

**Table 1:** Clinical characteristics of 82 AML patients treated with HSCT according to absolute pre-HSCT *BAALC*/*ABL1* copy numbers (high *vs*. low, 0.14 cut)

Characteristic	All patients (n=82)	Low pre-HSCT *BAALC/ABL1* copy numbers (n=61)	High pre-HSCT *BAALC/ABL1* copy numbers (n=21)	*P*
Pre-HSCT *BAALC*/*ABL1* copy numbers				<.001
Median	0.03	0.02	0.44	
Range	0.00-2.58	0.00-0.11	0.14-2.58	
Age at HSCT, years				.79
Median	63.9	64.9	63.9	
Range	50.8-76.2	51.5-76.2	50.8-74.9	
Sex, n (%)				.80
Male	37	27 (44)	10 (48)	
Female	45	34 (56)	11 (52)	
Hemoglobin at diagnosis, g/dL				.54
Median	8.7	9.0	8.5	
Range	4.5-14.4	5.5-14.4	4.5-11.3	
Platelet count at diagnosis, x 10^9^/L				.76
Median	65	71	63	
Range	3-224	3-167	13-224	
WBC count at diagnosis, x 10^9^/L				.13
Median	7.2	4.6	22.4	
Range	0.7-385	0.8-324	0.7-385	
Blood blasts at diagnosis, %				.48
Median	22	21	28	
Range	0-97	0-97	2-97	
BM blasts at diagnosis, %				.87
Median	50	52	43	
Range	3-95	3-95	10-95	
Karyotype, n (%)				.45
Abnormal	41	32 (55)	9 (43)	
Normal	38	26 (45)	12 (57)	
ELN 2010 Genetic Group, n (%) [36]				.86
Favorable	17	12 (22)	5 (26)	
Intermediate-I	19	13 (24)	6 (32)	
Intermediate-II	19	15 (27)	4 (21)	
Adverse	19	15 (27)	4 (21)	
Disease origin, n (%)				.60
*De novo*	52	40 (66)	12 (57)	
Secondary	30	21 (34)	9 (43)	
*NPM1* at diagnosis, n (%)				.76
Wild-type	51	36 (77)	15 (71)	
Mutated	17	11 (23)	6 (29)	
*FLT3*-ITD at diagnosis, n (%)				1
Absent	54	38 (79)	16 (80)	
Present	14	10 (21)	4 (20)	
*CEBPA* at diagnosis, n (%)				.09
Wild-type	51	34 (83)	17 (100)	
Mutated	7	7 (17)	0 (0)	

*ABL1*, Abelson murine leukemia viral oncogene homolog 1 gene; *BAALC*, brain and acute leukemia, cytoplasmatic gene; BM, bone marrow; *CEBPA*, CCAAT/enhancer-binding protein alpha gene; ELN, European LeukemiaNet classification 2010; *FLT3*-ITD, internal tandem duplication of the fms like tyrosine kinase 3 gene; HSCT, hematopoietic stem cell transplantation; *NPM1*, nucleophosmin 1 gene; WBC, white blood cell.

### Prognostic significance of pre-HSCT *BAALC/ABL1* copy numbers

**Figure 2:**
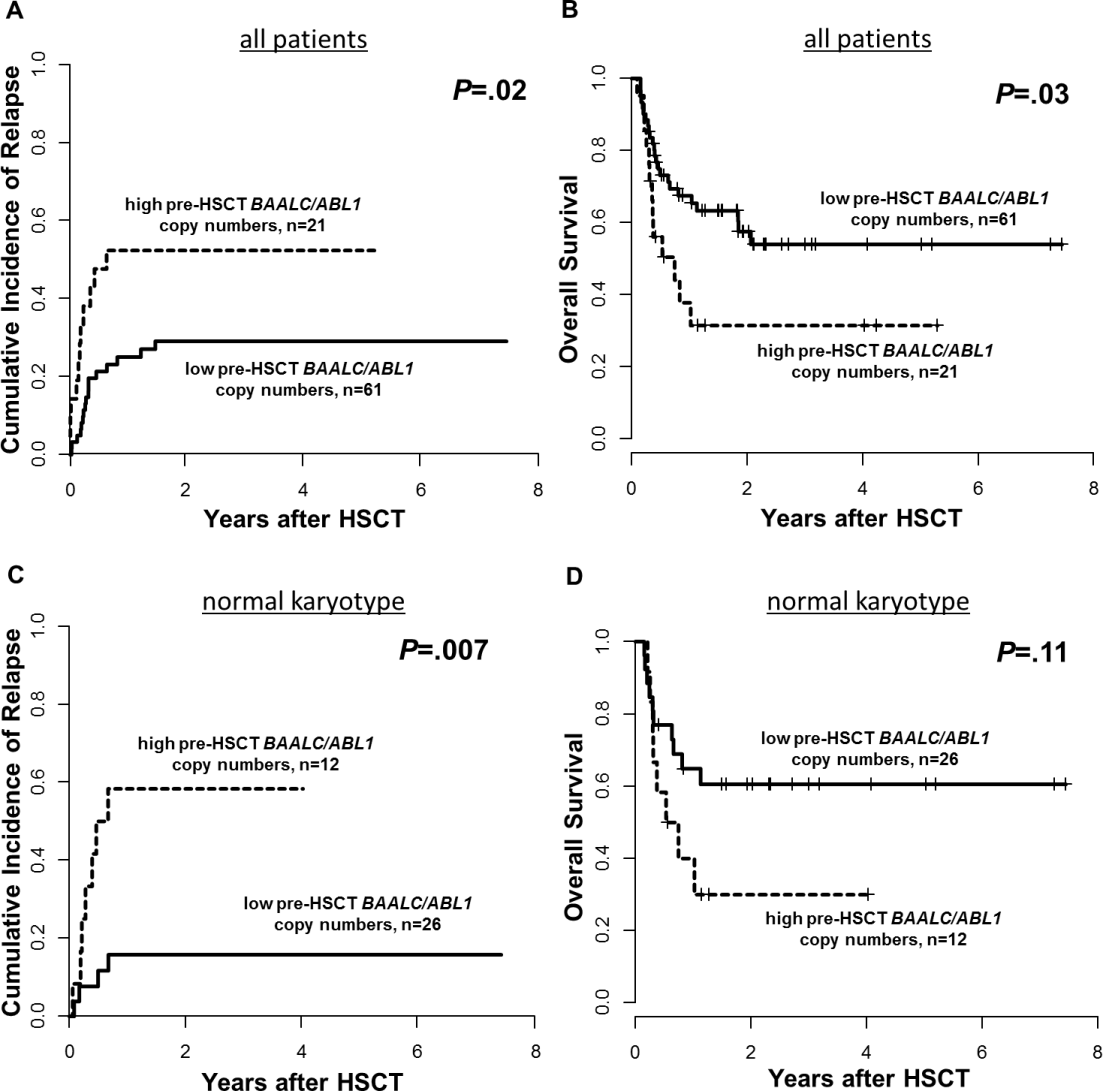
Outcome of patients according to pre-HSCT *BAALC*/*ABL1* copy numbers, high *vs* low, 0. 14 cut, **(A)** Cumulative Incidence of Relapse and **(B)** Overall Survival for the entire set (n=82) and **(C)** Cumulative Incidence of relapse and **(D)** Overall Survival in patients with a normal karyotype (n=38).

Patients with high pre-HSCT *BAALC*/*ABL1* copy numbers had a significantly higher cumulative incidence of relapse (CIR, *P*=.02, Figure 2A) and shorter overall survival (OS, *P*=.03, Figure 2B) which was reproduced when we restricted our analysis to patients with a normal karyotype (n=38, *P*=.007 and *P*=.11, respectively, Figures 2C and 2D). Subgroup analyses for patients harboring *de novo* disease (n=52, Supplementary Figure 2), patients transplanted in CR (n=68, Supplementary Figure 3), CD34-positive AML (n=31, Supplementary Figure 4), patients surviving longer than 100 days after HSCT (n=71, Supplementary Figure 5), as well as patients with diagnostic *BAALC*/*ABL1* copy number information available (n=51, Supplementary Figure 6) are shown in the Supplementary Materials.

**Figure 3:**
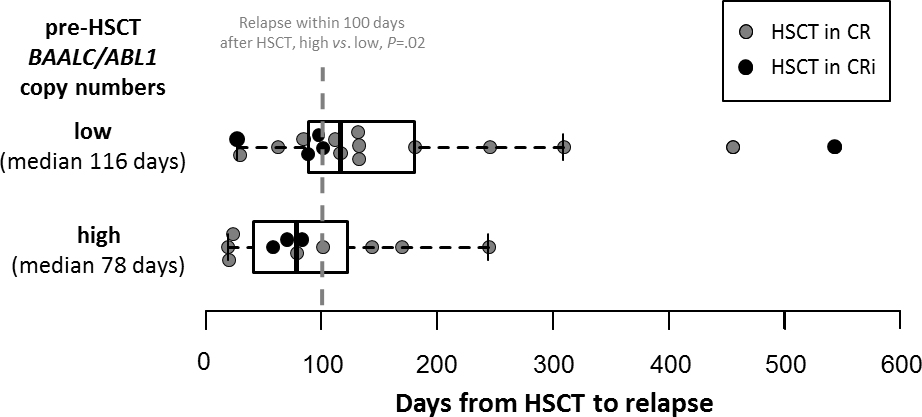
Time from HSCT to relapse according to high (median 78, range 19-244 days) or low (median 116, range 27-543 days) absolute pre-HSCT *BAALC*/*ABL1* copy numbers in relapsed patients (n=28)

One year after HSCT, 52% of patients with high pre-HSCT *BAALC*/*ABL1* copy numbers relapsed compared to 25% of patients with low pre-HSCT *BAALC*/*ABL1* copy numbers. Furthermore, 38% of patients with high pre-HSCT *BAALC*/*ABL1* copy numbers were alive compared to 68% of patients with low pre-HSCT *BAALC*/*ABL1* copy numbers. Patients with high pre-HSCT *BAALC*/*ABL1* copy numbers suffering relapse had a trend for shorter time to relapse after HSCT (median 78, range 19-244 days) compared to patients with low pre-HSCT *BAALC*/*ABL1* copy numbers (median 116, range 27-543 days, *P*=.07). Furthermore, for patients without non-relapse mortality after 100 days and six months after HSCT, those with high pre-HSCT *BAALC*/*ABL1* copy numbers more often relapsed compared to patients with low pre-HSCT *BAALC*/*ABL1* copy numbers (37% *vs*. 11%, *P*=.02 (Figure 3), and 73% *vs*. 27%, *P*=.002, respectively). In multivariable analysis, high pre-HSCT *BAALC*/*ABL1* copy numbers significantly associated with higher CIR (Hazard Ratio [HR] 2.6, Confidence Interval [CI] 1.2-5.7, *P*=.01) after adjustment for disease status at HSCT (*P*=.003) and disease origin (*P*=.009) and shorter OS (HR 2.1, CI 1.1-4.1, *P*=.03, Table 2).

**Table 2:** Multivariable outcome analyses of 82 AML patients treated with HSCT

Variable	Cumulative Incidence of Relapse		Overall survival	
	HR^a^ (95% CI)	*P*	HR^a^ (95% CI)	*P*
pre-HSCT *BAALC*/*ABL1* copy numbers (high *vs*. low, 0.14 cut)	2.6 (1.2-5.7)	.012	2.1 (1.1-4.1)	.03
Disease origin (*de novo vs*. secondary)	0.4 (0.2-0.8)	.009	-	-
Disease status at HSCT (CR *vs*. CRi)	0.3 (0.1-0.7)	.003	-	-

*ABL1*, Abelson murine leukemia viral oncogene homolog 1 gene; *BAALC*, brain and acute leukemia, cytoplasmatic gene; CI, confidence interval; CR, complete remission; CRi, CR with incomplete peripheral recovery; HSCT, hematopoietic cell transplantation; HR, hazard ratio.

^a^ HR, hazard ratio, <1 (>1) indicate lower (higher) risk for an event for the first category listed for the dichotomous variables.

Variables considered in the models were those significant at α=0.20 in univariable analyses. For OS endpoint, variables considered were hemoglobin count at diagnosis, white blood cell count at diagnosis, pre-HSCT *BAALC/ABL1* copy numbers (high vs. low) and HLA match (antigen match vs mismatch) while for CIR endpoint, variables considered were disease origin (de novo vs. secondary), *BAALC/ABL1* copy numbers (high vs. low), disease status at HSCT (CR vs. CRi) and ELN 2010 Genetic Group.

Detailed comparisons between the four groups of patients experiencing relapse or remaining in remission with high or low pre-HSCT *BAALC*/*ABL1* copy numbers are shown in the Supplementary Materials.

## DISCUSSION

Assessment of residual disease provides a powerful tool to measure treatment responses and to identify patients at high risk of relapse [4]. Although we still lack data of prospective MRD-guided trials in non-APL (acute promyelocyte leukemia) AML, MRD assessment may allow preemptive therapy to delay or even prevent relapse and improve outcomes [5, 26, 27]. However, about 40% of AML patients do not harbor the today commonly used molecular MRD targets [3, 4], reflecting a need for new MRD markers.

While the prognostic impact of high *BAALC* expression levels at diagnosis has been widely evaluated [3, 16–19], only a few studies with limited patient numbers evaluated *BAALC* expression levels during disease course using qRT-PCR [11, 15, 20, 21]. For MRD evaluation in AML in general, it remains unclear whether peripheral blood or bone marrow should be analyzed [7, 28]. For *BAALC*, high correlations of expression levels in peripheral blood and bone marrow in both newly diagnosed AML patients and healthy individuals have been shown [15, 16]. While some authors only used bone marrow [21], others used *BAALC* expression levels of either blood or bone marrow for survival analysis at diagnosis and during disease course [11, 20]. Peripheral blood is derived faster, with lower risk of complications and a higher convenience for the patient than bone marrow aspiration and results in comparable *BAALC* expression data [15, 16]. Therefore, we decided to restrict our analysis to peripheral blood samples to examine the prognostic impact of absolute pre-HSCT *BAALC*/*ABL1* copy numbers in patients receiving NMA-HSCT.

None of the aforementioned studies focusing on *BAALC* expression levels during disease course reported significant diagnostic clinical or genetic associations with different *BAALC* expression levels at a defined point in time in CR. In our study, we also did not detect any significant association of high pre-HSCT *BAALC*/*ABL1* copy numbers with tested pre-treatment or pre-HSCT parameters. This may indicate that the observed higher CIR and subsequent shorter OS is indeed driven by residual disease detected by high pre-HSCT *BAALC*/*ABL1* copy numbers rather than other commonly tested prognostic parameters. The first study to propose *BAALC* as a potential MRD marker analyzed 45 patients with *de novo* acute leukemia, but also included six patients with APL and 11 patients with lymphoid leukemia in their analysis [15]. The authors were able to show a superior disease-free survival in patients with lower *BAALC/GAPDH* expression levels in bone marrow after CR achievement. Another small study focused on 45 patients harboring core-binding factor (CBF) AML that received an allogeneic or autologous HSCT and evaluated *BAALC*/*ABL1* expression levels in bone marrow at diagnosis, as well as in CR after the first induction cycle, pre-HSCT, and at day 60 post-HSCT. While the authors showed significantly shorter OS, event-free survival (EFS) and higher CIR in patients with high *BAALC*/*ABL1* expression levels at diagnosis and post-HSCT, there was no significant impact on outcome after first induction cycle or pre-HSCT [21]. In contrast, we observed a strong prognostic impact of high pre-HSCT *BAALC*/*ABL1* copy numbers on CIR and OS in univariable and multivariable models. These differences might be explained by a lower patient number (n=45) and/or the restriction to CBF AML in the study of Yoon *et al*. [21]. In 27 CN-AML patients with high initial *BAALC*/*ABL1* expression levels, Weber *et al*. [11] observed shorter EFS for individuals with sustained high *BAALC*/*ABL1* expression levels in peripheral blood or bone marrow after two induction cycles. Later, this data was extended to 46 and 33 patients after completion of two induction cycles and 3-6 months after completion of two induction cycles, respectively [11]. Again, patients with high *BAALC*/*ABL1* expression levels at either of both time points had shorter EFS. Despite this promising data, possible limitations of *BAALC* as MRD marker still have to be determined. While most studies showed a prognostic impact without a prior assessment of the CD34 expression status [11, 20, 21], Najima *et al*. [15] postulated *BAALC* as MRD marker limited to CD34-positive AML as *BAALC* is upregulated in CD34-positive AML [14]. Restricting our analysis to patients with CD34-positive AML, we also observed a trend for higher CIR in patients with high pre-HSCT *BAALC*/*ABL1* copy numbers despite low patient numbers (*P*=.06, n=31, Supplementary Material). Limited numbers of patients with CD34-negative AML prevented a separate analysis for this subset. However, we observed no difference in CD34-positivity or CD34 expression at diagnosis between patients with high or low pre-HSCT *BAALC*/*ABL1* copy numbers (Supplementary Table 1). Further studies are needed to evaluate whether there are specific AML subgroups for which *BAALC* represents a more suitable MRD marker than for others.

To our knowledge until today all studies used qRT-PCR for *BAALC* evaluation but different approaches to define a cut-off for high or low *BAALC* expression levels during follow-up. Najima *et al*. [15] used the two-fold standard deviation over the median of a healthy cohort, while Yoon *et al*. [21] focused on the relative *BAALC*/*ABL1* expression of the tested patients and used a ROC (receiver operation characteristic) curve to define the optimal cut for each point in time individually. The latter resembles our approach and – despite different methodology - the evaluated cut-off in our cohort was also slightly higher than the two-fold standard deviation over the median of healthy *BAALC*/*ABL1* copy numbers in peripheral blood (0.14 vs. 0.10, respectively). Finally, Weber *et al*. used the median *BAALC*/*ABL1* expression at diagnosis of the initial cohort [11] to define high or low expression during disease course but restricted their analysis to patients with initially high *BAALC*/*ABL1* expression levels [11, 20]. In our study, for a subset of the analyzed patients (n=51) diagnostic material for *BAALC*/*ABL1* copy number assessment was available. For patients’ characteristic, as well as clinical and biological associations linked with high *BAALC*/*ABL1* copy numbers at diagnosis see the Supplementary Material. When we restricted our outcome analyses to patients with low or high *BAALC*/*ABL1* copy numbers at diagnosis - despite the limited number of patients - we observed a trend for higher CIR and shorter OS for patients with high pre-HSCT *BAALC*/*ABL1* copy numbers in patients irrespective of the diagnostic *BAALC*/*ABL1* copy number (Supplementary Figure 6). In fact five of the patients with low diagnostic *BAALC*/*ABL1* copy numbers had high pre-HSCT *BAALC*/*ABL1* copy numbers, of which three subsequently relapsed (see Supplementary Material for details). Thus, despite the limited number of patients, our data indicate that pre-HSCT *BAALC/ABL1* copy number determination can provide valuable clinical information also in patients with low diagnostic *BAALC*/*ABL1* copy numbers.

Considering the small number of studies focusing on *BAALC* expression as a MRD marker, the optimal cut-off needs validation. However, assessment of *BCR*-*ABL1* as MRD marker in CML showed us the technical difficulties of standard curves and in achieving an inter-laboratory comparability to ensure consistent analyses [22]. ddPCR has already been shown to provide comparable sensitivity to qRT-PCR but seems to have an improved day-to-day reproducibility and greater precision [23, 29, Huang et al, ASH 2015]. Therefore, ddPCR may represent a promising new method for gene expression analyses for MRD monitoring in the future.

Our here presented study is the first to demonstrate that ddPCR is a feasible method for evaluation of absolute *BAALC*/*ABL1* copy numbers prior to allogeneic HSCT. We were able to show that patients with high pre-HSCT *BAALC*/*ABL1* copy numbers had a significant higher CIR and shorter OS (*P*=.02 and *P*=.03, respectively, Figure 2). Patients with high pre-HSCT *BAALC*/*ABL1* copy numbers had an over 2.5-fold higher risk of relapse and an over 2-fold higher risk of death after HSCT compared to patients with low pre-HSCT *BAALC*/*ABL1* copy numbers (Table 2). Patients with high pre-HSCT *BAALC*/*ABL1* copy numbers more often suffered relapse within the first 100 days after HSCT (37% *vs*. 11%, *P*=.02) and the time from HSCT to relapse was shorter in patients with high pre-HSCT *BAALC*/*ABL1* copy numbers by trend (*P*=.07, Figure 3). To our knowledge, no other study reported on early relapses detected by high *BAALC* expression levels. We postulate that high pre-HSCT *BAALC*/*ABL1* copy numbers might indicate a residual disease burden in AML patients that subsequently may lead to early relapse during follow-up. Noteworthy, for all patients, peripheral blood was used in the analyses facilitating repetitive MRD assessment. We and others [11, 15, 20, 21] were able to show that *BAALC* has the potential to allow further risk stratification during disease course and subsequently may improve MRD assessment in addition to other established MRD markers such as *PML-RARA*, *CBFB-MYH11, RUNX1-RUNX1T1* or *NPM1* mutations. Furthermore, since *BAALC* is expressed at different amounts in all AML patients, it might allow molecular MRD detection in patients lacking molecular alterations commonly used for MRD assessment.

Restrictions of our study are the retrospective nature and the limited patient numbers. Future prospective clinical trials are needed to validate the here-established cut-off value and the resulting outcome findings in larger patient populations.

Even with a variety of possible treatment options such as reduction of immunosuppression, donor lymphocyte infusions or treatment with hypomethylating agents, patients suffering from morphologic relapse after HSCT have a very poor prognosis [25, 30, 31]. Pre-HSCT *BAALC*/*ABL1* copy number evaluation allows early identification of patients at higher risk of relapse and subsequently closer monitoring for relapse in the post-transplant period. In the future pre-HSCT *BAALC*/*ABL1* evaluation might guide preemptive treatment to improve the poor prognosis of AML patients with a risk for morphologic relapse. Furthermore, prospective studies will be required to evaluate whether AML patients with high pre-HSCT *BAALC*/*ABL1* copy numbers might benefit from additional treatment or intensification of the conditioning regimen prior to allogeneic HSCT.

## MATERIALS AND METHODS

### Patients and treatment

A total of 82 adult AML patients who received allogeneic HSCT at the University of Leipzig between September 2002 and December 2015 were retrospectively included in this analysis. All patients had peripheral blood samples up to 14 days prior to HSCT (median 7, range 0-14 days) for *BAALC*/*ABL1* copy number assessment available. White blood count (WBC) was assessed at time of blood sampling for analysis. Additionally, for 51 of these patients diagnostic peripheral blood or bone marrow samples were available for *BAALC*/*ABL1* copy number analysis. For details see Supplementary Materials and Supplementary Table 3.

All patients received age-dependent cytarabine based chemotherapy protocols (under or over 60 years) and were consolidated with HSCT in first (60%) or second CR (23%) or CRi (17%). For details please see Supplementary Materials. Median age at HSCT was 63.9 (range 50.8-76.2) years. Written informed consent for participation in these studies was obtained in accordance with the Declaration of Helsinki.

All patients received NMA conditioning with fludarabine 30 mg/m^2^ for three days followed by 2 Gy total body irradiation [32, 33] and infusion of granulocyte colony stimulating factor (G-CSF)-mobilized peripheral blood stem cells on day 0. Reasons for choosing a NMA protocol were age over 50 years for patients receiving unrelated HSCT (n=71) or age over 55 years for patients receiving related HSCT (n=11). Patients’ characteristics are shown in Table 1 and Supplementary Table 1. For Information regarding prevention and incidence of acute and chronic graft-versus-host disease see Supplementary Material. Median follow-up after HSCT for patients alive was 1.8 years.

### Healthy control cohort

In a control cohort of 7 healthy volunteers (median age of 62.7, range 39.6-82.0 years), absolute *BAALC*/*ABL1* copy numbers in peripheral blood were evaluated. Written informed consent was obtained for all healthy individuals; their characteristics are shown in Supplementary Table 2.

### Cytogenetic, moleculargenetic, and flow cytometric analyses

At diagnosis, cytogenetic analyses, the presence of internal tandem duplication in the *FLT3* gene (*FLT3*-ITD) as well as mutations in the *FLT3* tyrosine kinase domain (*FLT3*-TKD), *NPM1* and *CEBPA* genes were determined as previously described [34]. For details see Supplementary Material. For patients with material available, the CD34 and CD38 expression on mononuclear cells in bone marrow at diagnosis was determined as previously described [35].

### ddPCR assessment of *BAALC/ABL1* copy numbers

Absolute *BAALC* copy numbers were assessed using a specific ddPCR assay (BioRad, Hercules, California, USA; Assay ID: dHsaCPE5025566) according to manufacturer's specifications. Primers and probe sequences for *ABL1* copy number assessment (Biomers, Ulm, Germany) are shown in the Supplementary Material. ddPCR was performed on a QX100 platform (BioRad) and QuantaSoft software (Biorad) was used for raw data processing. With the droplet generator, each sample was divided into approximately 10,000 - 20,000 partitions (droplets). After PCR amplification (for details see Supplementary Material) the samples were placed into the droplet reader, where each droplet was read as positive or negative for the gene expression by issuing specific fluorescence signals (FAM and HEX). Redistribution according to the Poisson's algorithm determined the target copy number in the original sample. Two examples of the ddPCR droplet reader output are given in Supplementary Figure 1.

### *BAALC*/*ABL1* cut-off point definition

Using the R package ‘OptimalCutpoints’ a cut-off point of 0.1397 absolute pre-HSCT *BAALC*/*ABL1* copies was determined and used to define patients with high (n=21, 26%) and low (n=61, 74%) pre-HSCT *BAALC*/*ABL1* copy numbers in peripheral blood. For details see Supplementary Materials.

### End points and statistical analyses

For definition of clinical endpoints and statistical analyses for associations and survival (univariable and multivariable) see Supplementary Materials.

## SUPPLEMENTARY MATERIALS FIGURES, TABLES AND REFERENCES






