# Supplementary material for: High BAALC copy numbers in peripheral blood prior to allogeneic transplantation predict early relapse in acute myeloid leukemia patients
Source: Oncotarget. 2017 Sep 27;8(50):87944–54. doi: 10.18632/oncotarget.21322 (PMC5675684; doi:10.18632/oncotarget.21322)
Supplement: Supplementary file 1 [file oncotarget-08-87944-s001.pdf]

High *BAALC* copy numbers in peripheral blood prior to allogeneic transplantation predict early relapse in acute myeloid leukemia patients

SUPPLEMENTARY MATERIALS

Further genetic and HSCT related information

Additional cytogenetic and biological information and hematopoietic stem cell transplantation (HSCT) related information are shown in Supplementary Table 1.

Treatment protocols

Twenty-seven (93%) patients diagnosed with acute myeloid leukemia (AML) at the age of 60 years or younger received induction and consolidation therapy according to the AML 2002 study (#061) [1], and 2 (7%) patients received chemotherapy within the PKC412 protocol [2].

Among AML patients older than 60 years, 48 (90%) were treated within the AML 2004 (#069, ClinicalTrials.gov Identifier: NCT01497002) study [Niederwieser et al, ASH 2016], 3 (6%) patients were treated within the AML 2004 (#069) protocol and additionally received demethylating agents (azacitidine), 1 patient (2%) was treated within the OSHO #083 protocol and 1 (2%) patient was treated within the AMLCG 2008 (ClinicalTrials.gov Identifier: NCT01382147) protocol.

For consolidation therapy, all patients received an allogeneic HSCT after non-myeloablative conditioning (NMA) in complete remission (CR) or CR with incomplete peripheral recovery (CRi) at the University of Leipzig. Patients harboring a core-binding factor AML received an allogeneic HSCT in second CR or according to patients' choice in first CR if a suitable donor was available.

Prevention of graft-versus-host disease

All patients received a starting dose of 5 mg/kg body weight (BW) Ciclosporine A (CyA) in two daily doses from day -1. Blood levels of CyA were measured from day 0 and doses were adjusted for target levels of 200 ng/ml. Additionally, patients received mycophenolate mofetil (MMF) 3 g per day in three daily doses if receiving unrelated HSCT or 2 g per day in two daily doses if receiving related HSCT. CyA was reduced starting on day +84 or day +180 following related or unrelated HSCT, respectively, and MMF was stopped at day +28 following related HSCT and tapered from days +40 to +96 following unrelated HSCT [3].

For all patients after HSCT, immunosuppression was prolonged or extended with systemic steroids in cases of graft-versus-host disease (GvHD, grade > 2 according to Glucksberg grading system) [4] or rapidly reduced in patients who relapsed (≥ 5% blasts in bone marrow [BM]).

Incidence of GvHD

Patients were evaluated for incidence of acute GvHD (aGvHD) and chronic GvHD (cGvHD), using established criteria of the Glucksberg grading system [4]. Requirement for aGvHD was engraftment while requirements for cGvHD was engraftment and survival for at least 100 days after HSCT. There was no difference in development of aGvHD (grade 2-4) or cGvHD between patients with high or low pre-HSCT *BAALC*/*ABL1* copy numbers (*P*=.42 and *P*=.57, respectively, Supplementary Table 1).

ABL1 primers/probe and ddPCR information

ABL1 copy numbers were assessed using *ABL1* probe (ENP_1043: 5'-HEX-CCATTTTTGGTTTGGGCTTCACACCATT-BHQ1-3') with *ABL1* forward (ENF_1003: 5'-TTGAGATAACACTCTAAGCATAACTAAAGGT-3') and reverse (ENR_1063: 5'- GATGTAGTTGCTTGGGACCCA -3') primers (Biomers, Ulm, Germany).

The polymerase chain reaction (PCR) amplification was carried out at 95°C for 10 minutes (enzyme activation) followed by 40 PCR cycles at 94°C for 30 seconds (denaturation) and 56.6°C for 1 minute (annealing/extension) and a final 98°C for 10 minutes (enzyme deactivation). Supplementary Figure 1 gives two patient examples for the ddPCR droplet reader output.

Flow cytometry, cytogenetics, and further molecular markers

Pre-treatment bone marrow cytogenetic analyses were performed centrally in our institution using standard banding techniques. In cases were no metaphases could be obtained, fluorescence *in-situ* hybridization (FISH) was used to screen for recurrent abnormalities (i.e. del5/5q,

del7/7q, trisomy 8, inv(3), abn(p53), abn11q23, t(8;21), inv(16) and t(15;17); [n=3]).

Patients were grouped according to the European LeukemiaNet (ELN) 2010 classification in four risk groups [5].

Definition of complete remission

CR was defined as the presence of <5% of blasts in BM, neutrophils >1.0 x 10^9/L, platelets >100 x 10^9/L, absence of blasts with Auer rods, independence of blood transfusion and no extramedullary disease [5]. CRi was defined as CR with platelets <100 x 10^9/L or neutrophils <1.0 x 10^9/L. The presence of CR or CRi was confirmed within 28 days prior to HSCT by bone marrow and peripheral blood analysis.

Definition of clinical end points and statistical analysis

All statistical analyses were performed using the R statistical software platform (version 3.0.2). Cumulative incidence of relapse (CIR) was calculated from HSCT to morphologic relapse and overall survival (OS) was calculated from HSCT to death from any cause. Associations of the pre-HSCT *BAALC*/*ABL1* copy numbers with baseline clinical, demographic, and molecular features were compared using the Kruskal-Wallis test and Fisher's exact test for continuous and categorical variables, respectively. For OS, survival estimates were calculated using the Kaplan-Meier method and groups were compared with the log-rank test. CIR was calculated considering the competing risk (non-relapse mortality [NRM]) using the Fine and Gray model.

Survival of HSCT treated AML patients

The whole patient cohort had a 3-year OS rate of 48% (95% Confidence Interval [CI] 37-63%). CIR at 3 years after HSCT was 35% (CI 25-46%) and the competing event non-relapse mortality (NRM) at 3 years was 26% (CI 17-37%). This data is comparable with the existing literature reporting on similar non-myeloablative protocols [6–8].

Multivariable analysis

We constructed multivariable proportional hazard models for CIR and OS to evaluate the impact of pre-HSCT *BAALC*/*ABL1* copy numbers by forward adjusting for other variables.

In addition to pre-HSCT *BAALC*/*ABL1* copy numbers (high *vs.* low), the following variables were considered for multivariable analyses: sex, hemoglobin count, platelet count, white blood cell count (WBC), blast percentages in peripheral blood and BM at diagnosis, disease origin (*de novo vs.* secondary), ELN 2010 Genetic Group, age at HSCT, disease status at HSCT (CR *vs.* CRi), human leukocyte antigen (HLA) match (antigen match *vs.* mismatch), HLA donor type (related *vs.* unrelated) and sex of donor and recipient (female into male *vs.* all others). Of these, variables significant at α=.20 in univariable analyses were considered for multivariable analyses. For OS endpoint, these variables were hemoglobin count at diagnosis, white blood cell count at diagnosis, pre-HSCT *BAALC*/*ABL1* copy numbers (high *vs.* low) and HLA match (antigen match *vs.* mismatch) while for CIR endpoint, these variables were disease origin (*de novo vs.* secondary), *BAALC*/*ABL1* copy numbers (high *vs.* low), disease status at HSCT (CR *vs.* CRi) and ELN 2010 Genetic Group. Hazard ratios with their corresponding 95% confidence intervals were indicated for every significant prognostic factor.

Blood sampling for *BAALC/ABL1* copy number analysis

We observed no significant differences between the time from blood sampling for *BAALC*/*ABL1* copy number analysis or the WBC at blood sampling between patients with high or low pre-HSCT *BAALC*/*ABL1* copy numbers (Supplementary Table 1). There was also no correlation between WBC at blood sampling and pre-HSCT *BAALC*/*ABL1* copy numbers (Pearson correlation, R= -0.07).

Pre-HSCT *BAALC/ABL1* copy numbers and relapse

In the 61 patients with low pre-HSCT *BAALC*/*ABL1* copy numbers, 17 patients relapsed. In patients with low pre-HSCT *BAALC*/*ABL1* copy numbers, the housekeeper *ABL1* numbers did not differ significantly between patients suffering from relapse and patients remaining in remission (*P*=.85). Within the 21 patients with high pre-HSCT *BAALC*/*ABL1* copy numbers, 10 did not relapse. However, 5 of these 10 patients died from non-relapse mortality within 130 days and one additional patient 303 days after HSCT.

Analyzing the 28 patients that suffered from relapse separately, patients with high pre-HSCT *BAALC/ABL1* copy numbers (n=11) had significantly higher WBC (median 5.4 *vs.* 36 x 10^9/L, *P*=.05), higher blood blasts (median 10 *vs.* 37%, *P*=.03) and a higher CD34+/CD38- cell burden (median 0.2 *vs.* 5.8%, *P*=.03) at diagnosis and were more likely to harbor a normal karyotype (24 *vs.* 64%, *P*=.05) than patients with low pre-HSCT *BAALC/*

ABL1 copy numbers (n=17). Relapsing patients with high pre-HSCT *BAALC/ABL1* copy numbers also had a trend for shorter time from blood sampling to HSCT (median 7 *vs*. 6 days, *P*=.07) and for lower WBC at time of blood sampling for *BAALC/ABL1* copy number evaluation (median 5.2 *vs*. 2.6 x 10^9/L, *P*=.10). Analyzing the 54 patients that did not suffer from relapse separately, patients with high pre-HSCT *BAALC/ABL1* copy numbers (n=10) had a longer time from blood sampling to HSCT (median 7 *vs*. 8 days, *P*=.03) compared to patients with low pre-HSCT *BAALC/ABL1* copy numbers (n=44).

Analyzing the 21 patients with high pre-HSCT *BAALC/ABL1* copy numbers separately, patients suffering from relapse had a higher CD34+/CD38- cell burden (median 0.4 *vs*. 5.8, *P*=.05) and a trend for higher CD34 expression (median 5.6 *vs*. 44.8%, *P*=.10) at diagnosis as well as shorter time from blood sampling to HSCT (median 8 *vs*. 6 days, *P*=.03).

Analyzing the 61 patients with low pre-HSCT *BAALC/ABL1* copy numbers separately, patients suffering from relapse had a significantly lower platelet count at diagnosis (median 82 *vs*. 43 x 10^9/L, *P*=.03), less *de novo* disease (75 *vs*. 41%, *P*=.02) and were less likely to harbor a normal karyotype (54 *vs*. 24%, *P*=.05) or a *NPM1* mutation (31 *vs*. 0%, *P*=.05).

Prognostic value of pre-HSCT *BAALC/ABL1* copy numbers

Utilizing R's 'OptimalCutpoints' package [9] we identified a 0.1397 pre-HSCT *BAALC/ABL1* copy numbers cut-off to differentiate between patients suffering from relapse and patients remaining in remission. High pre-HSCT *BAALC/ABL1* copy numbers, defined using this cut-off value, also impacted on outcome when we restricted our analysis to patients diagnosed with *de novo* AML (by trend for CIR, *P*=.09 and significant for OS, *P*<.001, Supplementary Figure 2) or patients transplanted in CR (by trend for CIR, *P*=.08 and significant for OS, *P*=.01, Supplementary Figure 3). Low patient numbers prevented a separate analysis for patients transplanted in CRi (n=14). We observed no difference in NRM between patients with high or low pre-HSCT *BAALC/ABL1* copy numbers (whole cohort: *P*=.65, normal karyotype: *P*=.90, *de novo* AML: *P*=.18, patients transplanted in CR: *P*=.56)

CD34 expression at diagnosis

BAALC is known to be highly expressed in early CD34 positive bone marrow cells [10] and Najima *et al.* [11] postulated *BAALC* as measurable residual disease (MRD) marker in patients with CD34-positive AML. In our analysis, for 55 patients information on bone marrow CD34 positivity at diagnosis was available, 24 patients had CD34-negative and 31 patients CD34-positive AML. There was no significant difference of CD34 expression levels at diagnosis or CD34-positive disease in patients with high or low pre-HSCT *BAALC/ABL1* copy numbers (*P*=.55 and *P*=.57, respectively, Supplementary Table 1). Despite low patient numbers, we also observed a trend for shorter CIR (*P*=.06, Supplementary Figure 4) in patients with high pre-HSCT *BAALC/ABL1* copy numbers when we restricted our analysis to CD34-positive patients. Furthermore, there was a shorter time to relapse after HSCT (*P*=.04) in CD34-positive patients with high compared to CD34-positive patients with low pre-HSCT *BAALC/ABL1* copy numbers (median 64 [range 19-143] days and median 121 [range 29-543] days, respectively). However, with only 5 patients suffering from relapse with CD34-negative AML, no outcome analysis was possible for this subgroup.

Landmark analysis for patients surviving longer than 100 days after HSCT

To exclude possible aGvHD interaction effects, we performed a landmark analysis for the 71 patients that survived longer than 100 days after HSCT and also observed a significantly higher CIR (*P*=.03, Supplementary Figure 5A) and shorter OS (*P*=.04, Supplementary Figure 5B) for patients with high pre-HSCT *BAALC/ABL1* copy numbers.

BAALC/ABL1 copy numbers at diagnosis

For 51 of the 82 patients diagnostic bone marrow (n=46) or peripheral blood (n=5) samples were available. The median *BAALC/ABL1* copy number at diagnosis was 0.42 (range 0.01-7.10) which was significantly higher than the pre-HSCT *BAALC/ABL1* copy numbers (*P*<.001) or the *BAALC/ABL1* copy numbers in the healthy control (*P*=.003). The 0.14 cut-off was adopted and divided the cohort in patients with high (n=35, 69%) and low (n=16, 31%) *BAALC/ABL1* copy numbers at diagnosis. Patients' characteristics according to *BAALC/ABL1* copy numbers at diagnosis are displayed in Supplementary Table 3. Despite the low number of patients with material available, as described previously, high *BAALC/ABL1* copy numbers associated with a lower WBC by trend (*P*=.09) [12, 13] and a higher bone marrow CD34 expression (*P*<.001) [10] at diagnosis, a lower incidence of normal karyotype AML (*P*=.005) [14] and worse ELN 2010 genetic risk (*P*=.05). Patients with high *BAALC/ABL1* copy numbers were also more likely to be *NPM1* wild type (*P*<.001) [12–15] and to be *CEBPA* mutated by trend (*P*=.08) [12, 13, 15, 16]. High *BAALC/ABL1* copy numbers at diagnosis did not associate with higher pre-HSCT *BAALC/ABL1* copy numbers (*P*=.54).

We evaluated the prognostic impact of pre-HSCT *BAALC/ABL1* copy numbers in the two groups of patients with high or low *BAALC/ABL1* copy numbers at diagnosis separately. Despite the low patient numbers in these analyses, high pre-HSCT *BAALC/ABL1* copy numbers retained a trend for higher CIR and shorter OS in patients with low *BAALC/ABL1* copy numbers at diagnosis (n=16, Supplementary Figure 6A, 6B). Three of the five patients with high *BAALC/ABL1* copy numbers prior to HSCT relapsed and two patients died from HSCT-related causes less than 100 days after HSCT. Only two of eleven patients with low *BAALC/ABL1* copy numbers at diagnosis and low pre-HSCT *BAALC/ABL1* copy numbers suffered relapse. Thus, despite low patient numbers, our data indicate that pre-HSCT *BAALC/ABL1* copy numbers determination can provide valuable clinical information also in patients with low diagnostic *BAALC/ABL1* copy numbers. With chemotherapy, subclones might acquire resistance mechanism, expand over time and promote relapse genetically distinct from the AML at diagnosis [17–19]. Patients with low *BAALC* expression at diagnosis might suffer relapse from a clone with higher, and thus detectable *BAALC* expression during disease course.

SUPPLEMENTARY REFERENCES

1. Büchner T, Schlenk RF, Schaich M, Döhner K, Krahl R, Krauter J, Heil G, Krug U, Sauerland MC, Heinecke A, Späth D, Kramer M, Scholl S, et al. Acute Myeloid Leukemia (AML): different treatment strategies versus a common standard arm-combined prospective analysis by the German AML Intergroup. J Clin Oncol. 2012; 30:3604-3610.
2. Stone RM, Mandrekar SJ, Sanford BL, Laumann K, Geyer S, Bloomfield CD, Thiede C, Prior TW, Döhner K, Marcucci G, Lo-Coco F, Klisovic RB, Wei A, et al. Midostaurin plus Chemotherapy for Acute Myeloid Leukemia with a FLT3 Mutation. N Engl J Med. 2017. doi: 10.1056/NEJMoa1614359. [Epub ahead of print]
3. Niederwieser D, Maris M, Shizuru JA, Petersdorf E, Hegenbart U, Sandmaier BM, Maloney DG, Storer B, Lange T, Chauncey T, Deininger M, Pönisch W, Anasetti C, et al. Low-dose total body irradiation (TBI) and fludarabine followed by hematopoietic cell transplantation (HCT) from HLA-matched or mismatched unrelated donors and postgrafting immunosuppression with cyclosporine and mycophenolate mofetil (MMF) can induce durable complete chimerism and sustained remissions in patients with hematological diseases. Blood. 2003; 101:1620-1629.
4. Glucksberg H, Storb R, Fefer A, Buckner CD, Neiman PE, Clift RA, Lerner KG, Thomas ED. Clinical manifestations of graft-versus-host disease in human recipients of marrow from HLA matched sibling donors. Transplantation. 1974; 18:295-304.
5. Döhner H, Estey EH, Amadori S, Appelbaum FR, Büchner T, Burnett AK, Dombret H, Fenaux P, Grimwade D, Larson RA, Lo-Coco F, Naoe T, Niederwieser D, et al. Diagnosis and management of acute myeloid leukemia in adults: recommendations from an international expert panel, on behalf of the European LeukemiaNet. Blood. 2010; 115:453-474.
6. Walter RB, Gyurkocza B, Storer BE, Godwin CD, Pagel JM, Buckley SA, Sorror ML, Wood BL, Storb R, Appelbaum FR, Sandmaier BM. Comparison of minimal residual disease as outcome predictor for AML patients in first complete remission undergoing myeloablative or nonmyeloablative allogeneic hematopoietic cell transplantation. Leukemia. 2015; 29:137-144.
7. Versluis J, Labopin M, Niederwieser D, Socie G, Schlenk RF, Milpied N, Nagler A, Blaise D, Rocha V, Cornelissen JJ, Mohty M. Prediction of non-relapse mortality in recipients of reduced intensity conditioning allogeneic stem cell transplantation with AML in first complete remission. Leukemia. 2015; 29:51-57.
8. Gyurkocza B, Storb R, Storer BE, Chauncey TR, Lange T, Shizuru JA, Langston AA, Pulsipher MA, Bredeson CN, Maziarz RT, Bruno B, Petersen FB, Maris MB, et al. Nonmyeloablative allogeneic hematopoietic cell transplantation in patients with acute myeloid leukemia. J Clin Oncol. 2010; 28:2859-2867.
9. López-Ratón M, Rodríguez-Álvarez M, Cadarso-Suárez C, Gude-Sampedro F. OptimalCutpoints: An R Package for Selecting Optimal Cutpoints in Diagnostic Tests. *J Stat Software*. 2014; Volume 61, Issue 8.
10. Baldus CD, Tanner SM, Kusewitt DF, Liyanarachchi S, Choi C, Caligiuri MA, Bloomfield CD, de la Chapelle A. BAALC, a novel marker of human hematopoietic progenitor cells. Exp Hematol. 2003; 31:1051–1056.
11. Najima Y, Ohashi K, Kawamura M, Onozuka Y, Yamaguchi T, Akiyama H, Sakamaki H. Molecular monitoring of *BAALC* expression in patients with CD34-positive acute leukemia. Int J Hematol. 2010; 91:636–645.
12. Langer C, Radmacher MD, Ruppert AS, Whitman SP, Paschka P, Mrózek K, Baldus CD, Vukosavljevic T, Liu CG, Ross ME, Powell BL, de la Chapelle A, Kolitz JE, et al. High *BAALC* expression associates with other molecular prognostic markers, poor outcome, and a distinct gene expression signature in cytogenetically normal patients younger than 60 years with acute myeloid leukemia: a Cancer and Leukemia Group B (CALGB) study. Blood. 2008; 111:5371-5379.
13. Metzeler KH, Dufour A, Benthaus T, Hummel M, Sauerland MC, Heinecke A, Berdel WE, Büchner T, Wörmann B, Mansmann U, Braess J, Spiekermann K, Hiddemann W,

et al. *ERG* Expression Is an Independent Prognostic Factor and Allows Refined Risk Stratification in Cytogenetically Normal Acute Myeloid Leukemia: A Comprehensive Analysis of *ERG*, *MN1*, and *BAALC* Transcript Levels Using Oligonucleotide Microarrays. J Clin Oncol. 2009; 27:5031-5038.

14. Haferlach C, Kern W, Schindela S, Kohlmann A, Alpermann T, Schnittger S, Haferlach T. Gene expression of *BAALC*, *CDKN1B*, *ERG*, and *MN1* adds independent prognostic information to cytogenetics and molecular mutations in adult acute myeloid leukemia. Genes Chromosomes Cancer. 2012; 51:257-65.
15. Schwind S, Marcucci G, Maharry K, Radmacher MD, Mrózek K, Holland KB, Margeson D, Becker H, Whitman SP, Wu YZ, Metzeler KH, Powell BL, Kolitz JE, et al. *BAALC* and *ERG* expression levels are associated with outcome and distinct gene and microRNA expression profiles in older patients with de novo cytogenetically normal acute myeloid leukemia: a Cancer and Leukemia Group B study. Blood. 2010; 116:5660-5669.
16. Weber S, Alpermann T, Dicker F, Jeromin S, Nadarajah N, Eder C, Fasan A, Kohlmann A, Meggendorfer M, Haferlach C, Kern W, Haferlach T, Schnittger S. *BAALC* expression: a suitable marker for prognostic risk stratification and detection of residual disease in cytogenetically normal acute myeloid leukemia. Blood Cancer J. 2014;4:e173.
17. Döhner H, Weisdorf DJ, Bloomfield CD. Acute Myeloid Leukemia. N Engl J Med. 2015; 373:1136-52.
18. Ding L, Ley TJ, Larson DE, Miller CA, Koboldt DC, Welch JS, Ritchey JK, Young MA, Lamprecht T, McLellan MD, McMichael JF, Wallis JW, Lu C, et al. Clonal evolution in relapsed acute myeloid leukaemia revealed by whole-genome sequencing. Nature. 2012; 481:506-510.
19. Grimwade D, Ivey A, Huntly BJ. Molecular landscape of acute myeloid leukemia in younger adults and its clinical relevance. Blood. 2016; 127:29-41.

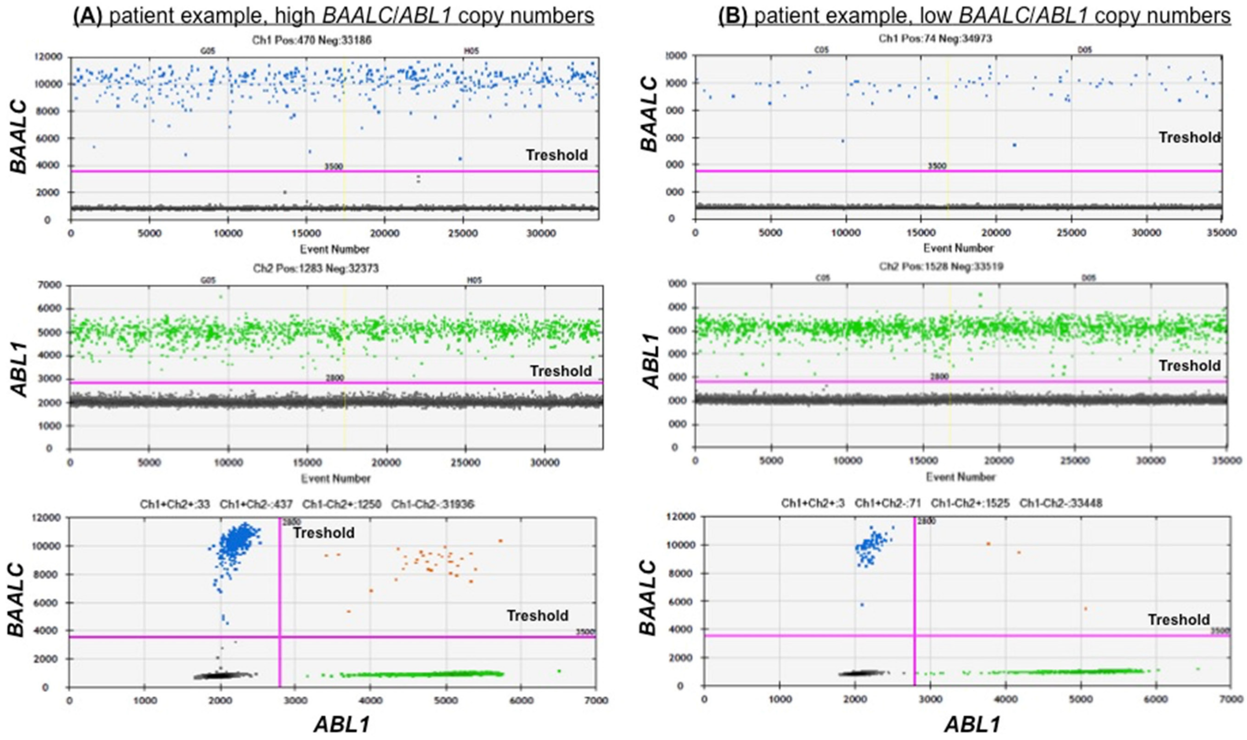

Supplementary Figure 1: Examples for ddPCR droplet reader output for a patient with **(A)** high *BAALC*/*ABL1* copy numbers (0.36 *BAALC*/*ABL1*) or **(B)** low *BAALC*/*ABL1* copy numbers (0.05 *BAALC*/*ABL1*).

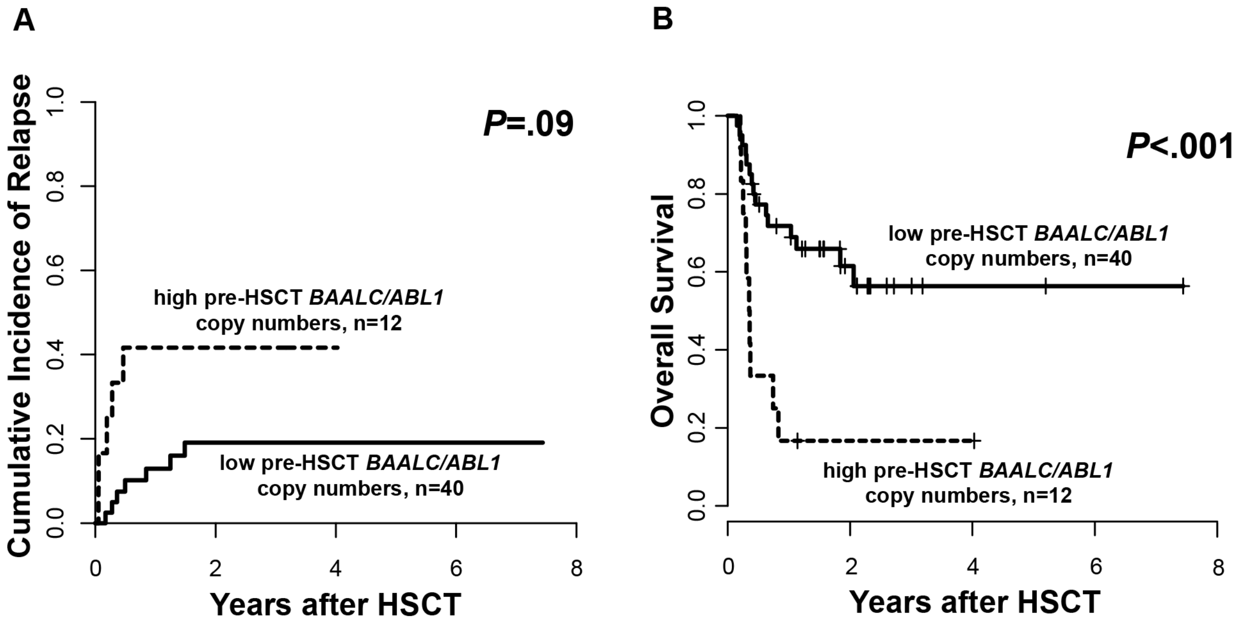

Supplementary Figure 2: Outcome of patients with *de novo* AML according to pre-HSCT *BAALC/ABL1* copy numbers, high *vs.* low, 0.14 cut, n=52. **(A)** Cumulative Incidence of Relapse and **(B)** Overall Survival.

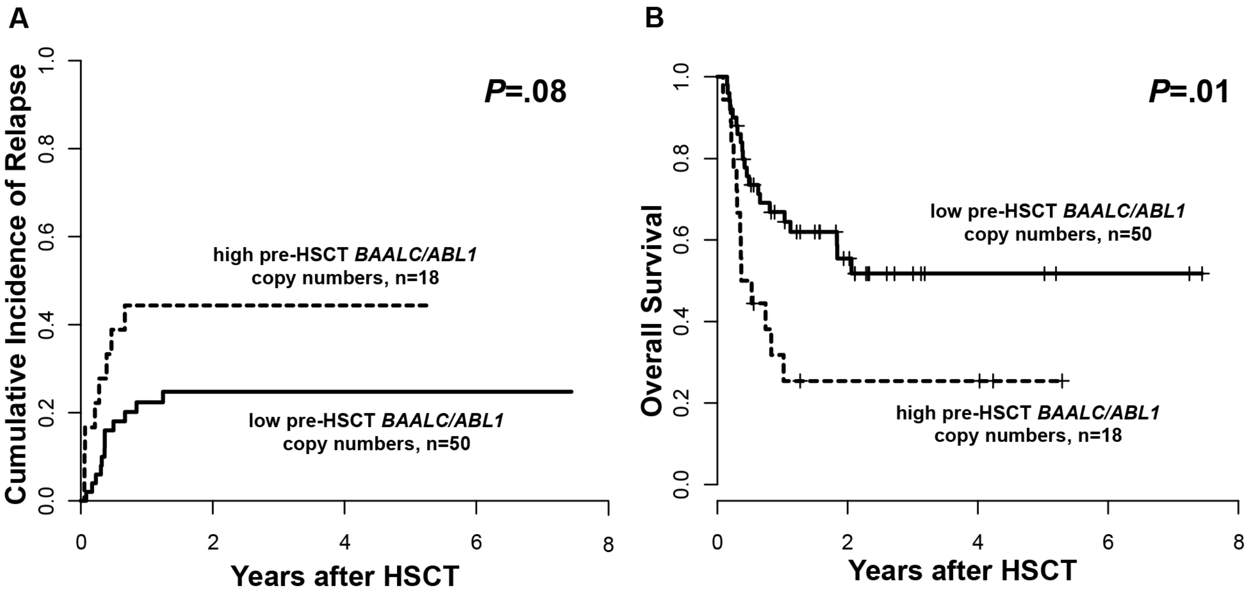

Supplementary Figure 3: Outcome of patients transplanted in CR according to pre-HSCT *BAALC*/*ABL1* copy numbers, high *vs.* low, 0.14 cut, n=68. **(A)** Cumulative Incidence of Relapse and **(B)** Overall Survival.

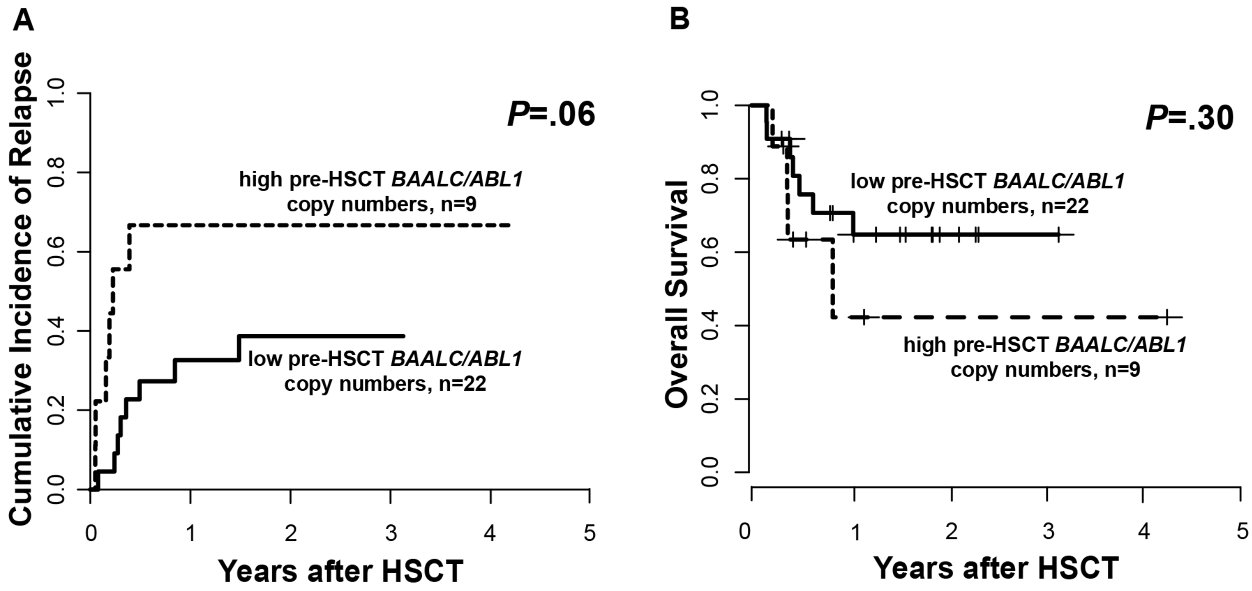

Supplementary Figure 4: Outcome of patients with CD34-positive AML according to pre-HSCT *BAALC*/*ABL1* copy numbers, high *vs.* low, 0.14 cut, n=31. **(A)** Cumulative Incidence of Relapse and **(B)** Overall Survival.

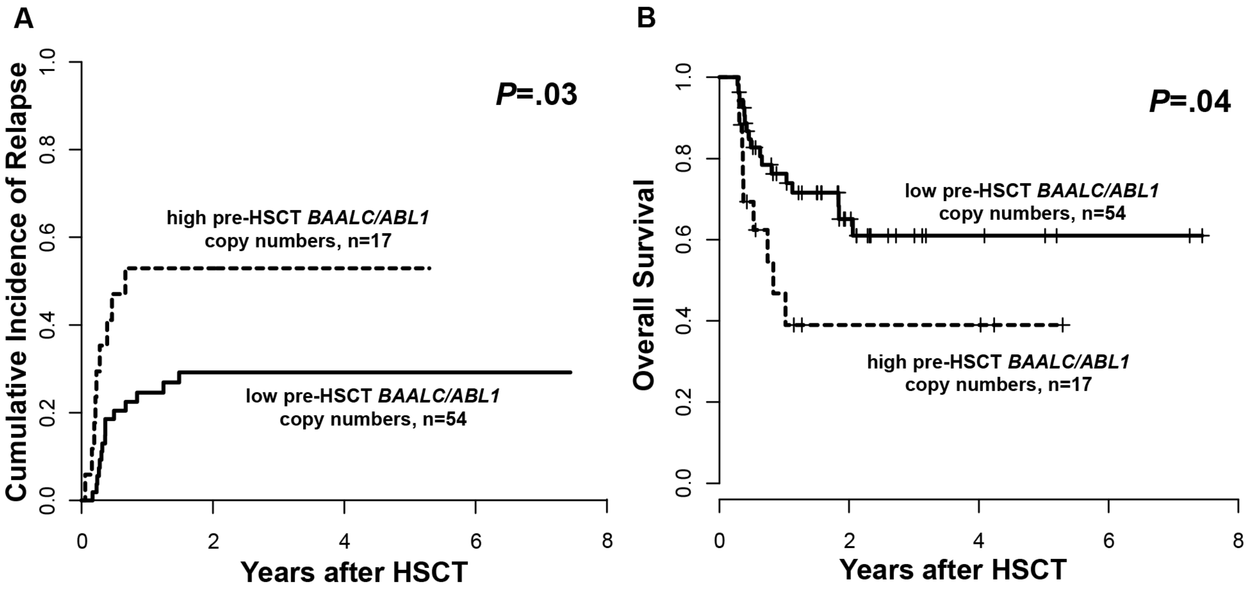

Supplementary Figure 5: Landmark analysis for patients surviving longer than 100 days after HSCT according to pre-HSCT *BAALC/ABL1* copy numbers, high *vs.* low, 0.14 cut, n=71. **(A)** Cumulative Incidence of Relapse and **(B)** Overall Survival.

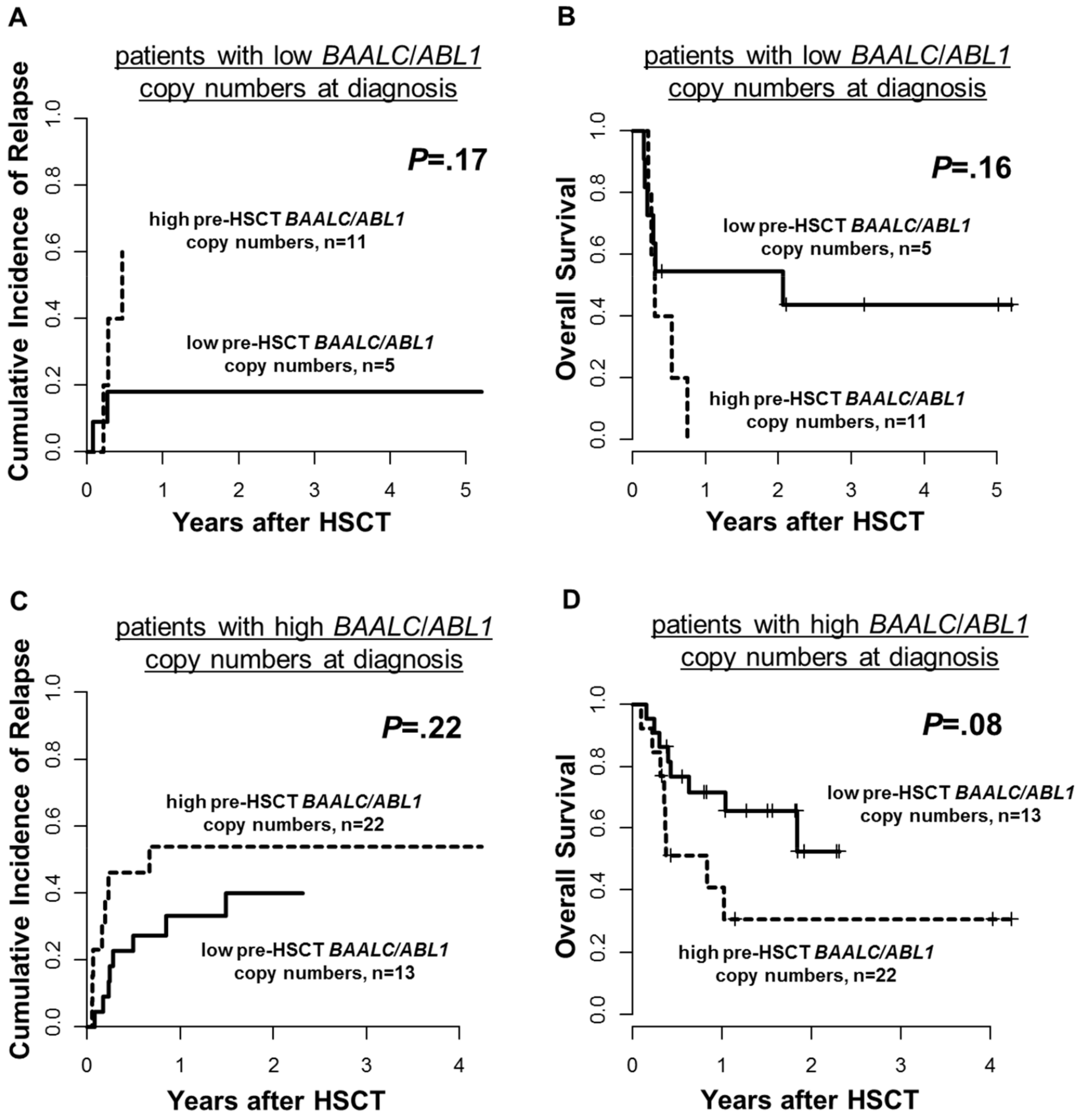

Supplementary Figure 6: Outcome of patients with high or low *BAALC/ABL1* copy numbers at diagnosis (0.14 cut) according to pre-HSCT *BAALC/ABL1* copy numbers, high *vs.* low, 0.14 cut. **(A)** Cumulative Incidence of Relapse and **(B)** Overall Survival in patients with low *BAALC/ABL1* copy numbers at diagnosis (n=16) and **(C)** Cumulative Incidence of Relapse and **(D)** Overall Survival in patients with high *BAALC/ABL1* copy numbers at diagnosis (n=35).

Supplementary Table 1: Additional clinical characteristics of HSCT treated AML patients according to absolute pre-HSCT *BAALC/ABL1* copy numbers, n=82

See Supplementary File 1

Supplementary Table 2: Comparison of AML patients (n=82) and the healthy control cohort (n=7)

Characteristic	Patients (n=82)	Healthy cohort (n=7)	*P*
Age, years			1
Median	63.9	62.7	
Range	50.8-76.2	39.6-82.0	
Sex, n (%)			1
Male	37	3	
Female	45	4	
BAALC/ABL1 copy numbers			.34
Median	0.03	0.04	
Range	0.00-2.58	0.03-0.10	

ABL1, Abelson murine leukemia viral oncogene homolog 1 gene; *BAALC*, brain and acute leukemia, cytoplasmatic gene.

Supplementary Table 3: Clinical characteristics of 51 AML patients with available material at diagnosis according to absolute *BAALC*/*ABL1* copy numbers at diagnosis (high *vs.* low, 0.14 cut)

See Supplementary File 2
