# Supplementary material for: High BAALC copy numbers in peripheral blood prior to allogeneic transplantation predict early relapse in acute myeloid leukemia patients
Source: Oncotarget. 2017 Sep 27;8(50):87944–54. doi: 10.18632/oncotarget.21322 (PMC5675684; doi:10.18632/oncotarget.21322)
Supplement: Supplementary file 2 [file oncotarget-08-87944-s002.docx]

**Supplementary Table 1:** **Additional clinical characteristics of HSCT treated AML patients according to absolute pre-HSCT *BAALC*/*ABL1* copy numbers, n=82**

| **Characteristic** | **All patients**  **(n=82)** | | **Low pre-HSCT *BAALC*/*ABL1* copy numbers (n=61)** | **High pre-HSCT *BAALC*/*ABL1* copy numbers (n=21)** | ***P*** |
| --- | --- | --- | --- | --- | --- |
| **Cytogenetic and molecular genetic information at diagnosis** | | | | | |
| Monosomal karyotype, n (%)  Absent  Present | 70  8 | | 52 (91)  5 (9) | 18 (86)  3 (14) | .67 |
| Complex karyotype, n (%)  Absent  Present | 67  11 | | 48 (84)  9 (16) | 19 (90)  2 (10) | .72 |
| Trisomy 8, n (%)  Absent  Present | 68  11 | | 48 (83)  10 (17) | 20 (95)  1 (5) | .27 |
| -5/del(5q), n (%)  Absent  Present | 72  8 | | 52 (88)  7 (12) | 20 (95)  1 (5) | .67 |
| -7/del(7q), n (%)  Absent  Present | 72  9 | | 53 (88)  7 (12) | 19 (90)  2 (10) | 1 |
| CBF AML, n (%)  Absent  Present | 77  2 | | 57 (98)  1 (2) | 20 (95)  1 (5) | .46 |
| *FLT3*-TKD, n (%)  Wild-type  Mutated | 58  5 | | 42 (95)  2 (5) | 16 (84)  3 (16) | .16 |
| **Flow cytometry at diagnosis** | | | | | |
| BM CD34 expression on mononuclear cells, %  Median  Range | 30  0.2-97 | | 31  0.2-90 | 20  0.2-97 | .55 |
| CD34-positive AML, n (%)  Absent  Present | 24  31 | | 15 (41)  22 (59) | 9 (50)  9 (50) | .57 |
| BM CD34+/CD38- expression on mononuclear cells, %  Median  Range | 0.5  0.0-63.0 | | 0.3  0.0-63.0 | 1.3  0-21.0 | .26 |
| **HSCT related information** | | | | | |
| Time from blood sampling to HSCT, days  median  range | | 7  0-14 | 7  2-14 | 7  0-11 | .63 |
| WBC at blood sampling, x 10^9^/L  median  range | | 4  0.4-10.6 | 4.2  0.4-10.6 | 3.6  0.5-8.9 | .31 |
| Remission at HSCT, n (%)  CR1  CR2  CRi | | 49  19  14 | 34 (56)  16 (26)  11 (18) | 15 (71)  3 (14)  3 (14) | .49 |
| HLA Antigen, n (%)  Match  Mismatch | | 58  24 | 42 (69)  19 (31) | 16 (76)  5 (24) | .59 |
| Donor, n (%)  Related  Unrelated | | 11  71 | 9 (15)  52 (85) | 2 (10)  19 (90) | .14 |
| Donor sex, n (%)  No female into male  Female into male | | 70  12 | 51 (84)  10 (16) | 19 (90)  2 (10) | .72 |
| aGvHD, n (%)  Absent  Present | | 42  25 | 31 (66)  16 (34) | 11 (55)  9 (45) | .42 |
| cGvHD, n (%)  Absent  Limited  Extended | | 14  8  29 | 12 (30)  7 (18)  21 (53) | 2 (18)  1 (9)  8 (73) | .57 |

*aGvHD, acute graft-versus-host disease; BM, bone marrow; CBF, core binding factor; cGvHD, chronic graft-versus-host disease; CR, complete remission; CRi, CR with incomplete peripheral recovery; FLT3-TKD, tyrosine kinase domain mutation in the FLT3 gene; HLA, human leukocyte antigen; HSCT, allogeneic hematopoietic stem cell transplantation; WBC, white blood count.*
