# Supplementary material for: High BAALC copy numbers in peripheral blood prior to allogeneic transplantation predict early relapse in acute myeloid leukemia patients
Source: Oncotarget. 2017 Sep 27;8(50):87944–54. doi: 10.18632/oncotarget.21322 (PMC5675684; doi:10.18632/oncotarget.21322)
Supplement: Supplementary file 3 [file oncotarget-08-87944-s003.docx]

**Supplementary Table 3: Clinical characteristics of 51 AML patients with available material at diagnosis according to absolute *BAALC*/*ABL1* copy numbers at diagnosis (high *vs.* low, 0.14 cut)**

| **Characteristic** | **All patients (n=51)** | **Low**  ***BAALC/ABL1* copy numbers at diagnosis (n=16)** | **High**  ***BAALC/ABL1* copy numbers at diagnosis (n=35)** | ***P*** |
| --- | --- | --- | --- | --- |
| *BAALC/ABL1 copy numbers at diagnosis*  *Median*  *Range* | 0.42  0.01-7.10 | 0.06  0.01-0.13 | 0.90  0.15-7.10 | <.001 |
| Age at diagnosis, years  Median  Range | 61.9  49.6-75.8 | 60.6  51.0-73.7 | 61.9  49.6-75.8 | .76 |
| Sex, n (%)  Male  Female | 21  30 | 6 (38)  10 (63) | 15 (43)  20 (57) | .77 |
| Hemoglobin at diagnosis, g/dL  Median  Range | 9.0  4.5-14.4 | 9.0  4.5-14.4 | 8.9  5.4-12.8 | .85 |
| Platelet count at diagnosis, x 10^9^/L  Median  Range | 65  13-224 | 80  14-207 | 63  13-224 | .73 |
| WBC count at diagnosis, x 10^9^/L  Median  Range | 8.8  0.8-385 | 32.3  0.8-324 | 6.9  0.9-385 | .09 |
| Blood blasts at diagnosis, %  Median  Range | 28  0-97 | 39  0-97 | 26  1-97 | .92 |
| BM blasts at diagnosis, %  Median  Range | 53.5  3-95 | 52  3-95 | 55  10-95 | .72 |
| BM CD34 expression at diagnosis, %  Median  range | 36.3  0.2-97 | 0.5  0.2-66 | 53.5  0.5-97 | <.001 |
| Karyotype, n (%)  Abnormal  Normal | 25  24 | 3 (20)  12 (80) | 22 (65)  12 (35) | .005 |
| ELN 2010 Genetic Group, n (%)[36]  Favorable  Intermediate-I  Intermediate-II  Adverse | 13  11  12  12 | 7 (47)  5 (33)  1 (7)  2 (13) | 6 (18)  6 (18)  11 (33)  10 (30) | .05 |
| Disease origin, n (%)  *De novo*  Secondary | 16  35 | 5 (31)  11 (69) | 11 (31)  24 (69) | 1 |
| *NPM1* at diagnosis, n (%)  Wild-type  Mutated | 38  13 | 5 (31)  11 (69) | 33 (94)  2 (6) | <.001 |
| *FLT3*-ITD at diagnosis, n (%)  Absent  Present | 39  11 | 12 (80)  3 (20) | 27 (77)  8 (23) | 1 |
| *CEBPA* at diagnosis, n (%)  Wild-type  Mutated | 37  6 | 15 (100)  0 (0) | 22 (79)  6 (21) | .08 |
| *Pre-HSCT BAALC/ABL1 copy numbers*  *Median*  *Range* | 0.05  0.00-2.58 | 0.05  0.00-0.96 | 0.04  0.00-2.58 | .54 |

*ABL1, Abelson murine leukemia viral oncogene homolog 1 gene; BAALC, brain and acute leukemia, cytoplasmatic gene; BM, bone marrow; CEBPA, CCAAT/enhancer-binding protein alpha gene; ELN, European LeukemiaNet classification 2010; FLT3-ITD, internal tandem duplication of the fms like tyrosine kinase 3 gene; HSCT, hematopoietic stem cell transplantation; NPM1, nucleophosmin 1 gene; WBC, white blood cell*
